# Supplementary material for: Genome-wide mutational signatures in low-coverage whole genome sequencing of cell-free DNA
Source: Nat Commun. 2022 Aug 23;13:4953. doi: 10.1038/s41467-022-32598-1 (PMC9399180; doi:10.1038/s41467-022-32598-1)
Supplement: Supplementary file 1 — Supplementary Information [file 41467_2022_32598_MOESM1_ESM.pdf]

# Genome-wide mutational signatures in low-coverage whole genome sequencing of cell-free DNA – Supplementary Information

Jonathan C. M. Wan[1], Dennis Stephens[1], Lingqi Luo[1], James R. White[1,2], Caitlin M. Stewart[1], Benoît Rousseau[1], Dana W. Y. Tsui[1,3], Luis A. Diaz, Jr.[1]*

**Affiliations:**

[1]Division of Solid Tumor Oncology, Memorial Sloan Kettering Cancer Center, New York, NY 10065, USA

[2]Resphera Biosciences, Baltimore, MD 21231, USA

[3]Current affiliation: Meyer Cancer Center, Weill Cornell Medical College, New York, NY, 10065, USA; New York Genome Center, New York, NY, 10013, USA

[4]Department of Pathology, Memorial Sloan Kettering Cancer Center, New York, NY 10065, USA

[5]Current affiliation: PetDx Inc., San Diego, USA

*Correspondence: diazl5@mskcc.org

## Supplementary Methods

*Individual SBS signature spike-in for sensitivity and specificity of signature fitting*

To assess the sensitivity of signature fitting to Pointy data, we performed an *in silico* signature spiking experiment. Varying numbers of mutations belonging to an individual SBS signature (10, 100 or 1,000) were iteratively spiked into a randomly selected healthy mutation profile. One signature at a time was spiked in, to assess the recovery of each signature using signature fitting, and was repeated for each SBS in Alexandrov et al.[1]. This was iteratively performed 100 times. In each iteration, prior to signature fitting, a background-subtraction step was performed, as described in the Methods.

A custom R script was used to generate spike-in signatures belonging to one signature only with a specified total number of mutations in the signature, utilizing countsSampling from the scRecover() package (v1.10.0). Fixed doses of specific mutational signatures were generated by sampling 10, 100 or 1,000 mutations (Supplementary Fig. 5b) with sampling frequencies equal to their frequency in reference mutational signatures[3].

The contribution of each signature was assessed pre- and post- spike. This allowed the calculation of sensitivity and specificity of signature fitting based on the ratio of observed vs. expected increase in mutations. The expected number of mutations per signature equals either the 10, 100 or 1,000 mutations that were spiked in, plus those originally present in the control sample.

The factors influencing signature fitting sensitivity were assessed by comparing cosine similarity between signatures and flatness, as determined by the standard deviation of the proportion contributions of each SBS context within the signature.

*Multiple SBS signature spike-in analysis*

To assess the performance of signature recovery in the setting of multiple signatures, we iteratively spiked in signatures, and simultaneously spiked in SBS1, at a ratio of 1:1 or 10:1 mutations. First, 10 mutations belonging to each SBS signature were spiked into a randomly selected control SBS profile, generated with the scRecover package, as above.

Then, either 10 or 100 mutations belonging to SBS1 were generated and spiked in. Recovery of each spiked signature was assessed, as before, with 100 iterations and background-subtraction. Significance testing was performed to assess change in sensitivity of spike in, using a Wilcoxon test with correction for multiple testing using the Benjamini-Hochberg method.

*Mutational signature profiling and detection in targeted sequencing*

Mutation calls (n = 170) generated by targeted sequencing were obtained for samples from the Georgiadis et al.[2] cohort (n = 15). Mutation calls were annotated with sequence context to generate a 96-SBS mutation profile for each sample. As mutation calls were already determined to be non-germline and above background noise, signatures were fitted directly, and a signature was detected if it had greater than zero mutations attributed to it.

*Classification across studies*

We tested Pointy using random forest (RF) classification for cancer detection across cohorts, as patients with CRC were common to both studies examined. All healthy individuals were included from the PGDX cohort (n = 19), and an equal number were randomly selected from the DELFI cohort (n = 19), sampled with a fixed seed. We identified evidence of batch effect affecting SNP-subtracted mutation profiles of healthy controls between the two studies (Supplementary Fig. 16a). Therefore, healthy and CRC patient samples were pooled across the two studies to enable training across batches. 10-fold nested CV was performed using RF with similar settings to before, repeated 10 times.

## Supplementary Figures

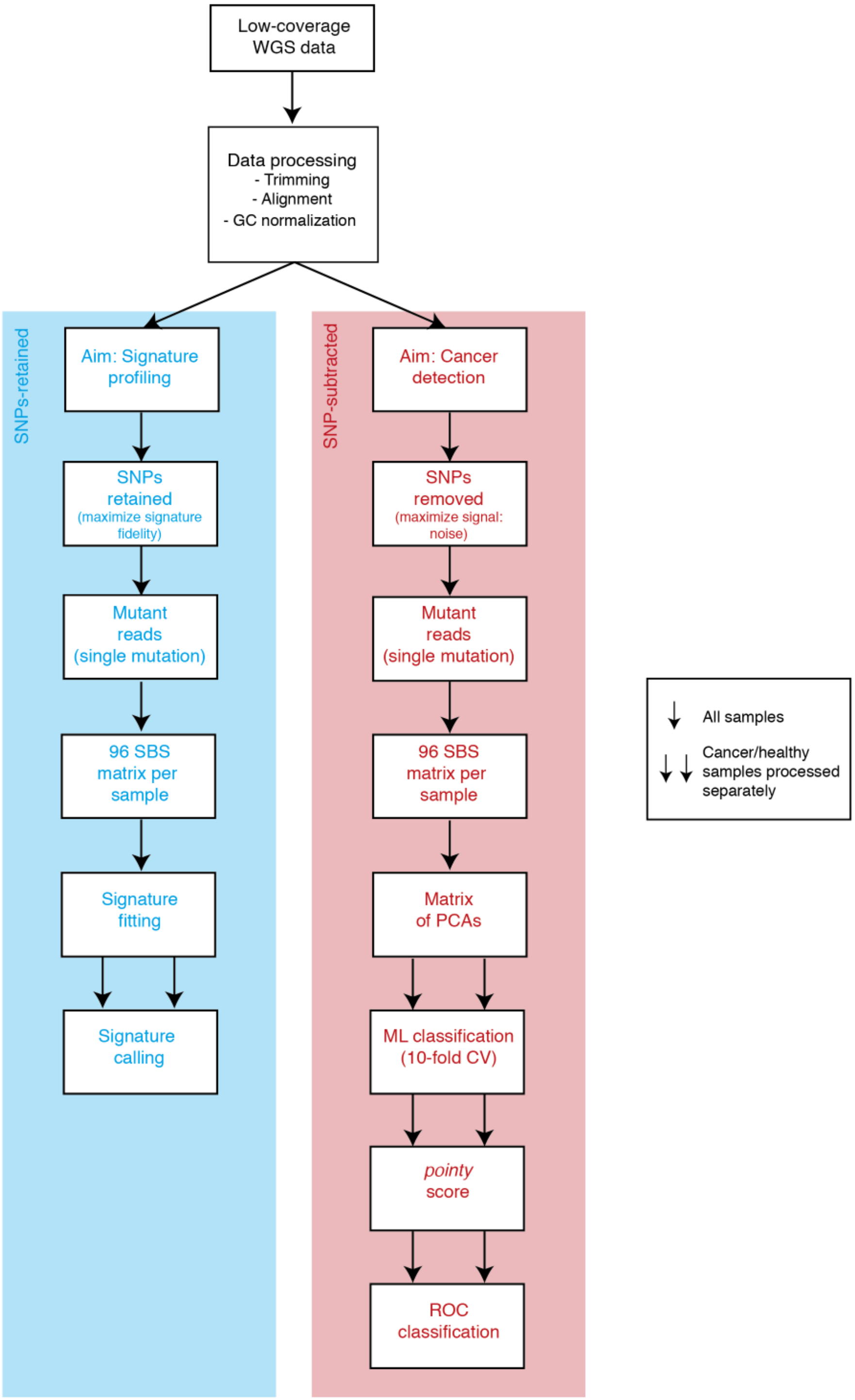


**Supplementary Fig. 1. Flow diagram of the Pointy pipeline.** This diagram shows the flow of samples through the pipeline depending on the purpose of the analysis. For all

samples, whole-genome sequencing (WGS) data were trimmed, aligned and GC-normalized (Methods). For mutational signature profiling, SNPs were retained in the data (blue), as bulk removal can distort the signature profile (Supplementary Fig. 10). For classification of samples as either cancer or healthy, SNPs were subtracted to maximize the signal-to-noise ratio (red). For both approaches, individual mutant reads were selected, which were used to generate a matrix of 96-SBS contexts per sample. Signatures were then fitted to each 96-SBS matrix for signature profiling. For sample classification, SNP-subtracted data were processed into principal components (PC) using principal component analysis (PCA), and PCs used as input for a classification model. A random forest model was used for classification, which generates a Pointy score for each sample, ranging from 0 to 1 (Supplementary Data 1). Pointy scores were used for classification with a threshold of 95% specificity (Methods). SNP, single nucleotide polymorphism; SBS, single base substitution; CV, cross-validation; ROC, receiver operating characteristic.

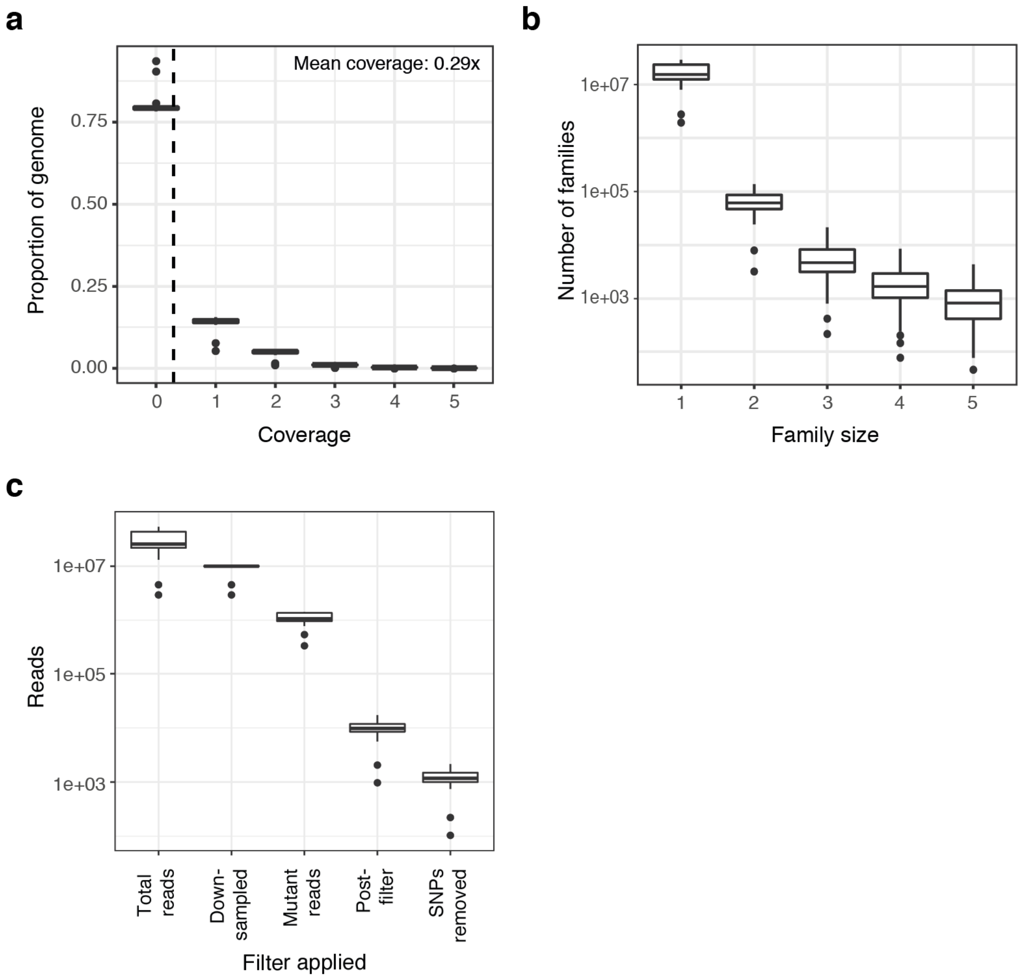


**Supplementary Fig. 2. Characterizing low-coverage plasma WGS data. (a)** PGDX plasma WGS samples (n = 37) were sequenced with a median of 31.0 x $10^6$ reads (15.5 x $10^6$ read families). Boxplots show the distribution of sequencing coverage following downsampling of sequencing reads to 10M reads (mean coverage = 0.29x). Boxplots represent median, bottom and upper quartiles, and the whiskers correspond to 1.5× IQR. The dashed vertical line indicates the mean coverage. (**b**) Boxplots showing the number of read families present for each read family size (of duplicates) in PGDX plasma WGS samples (n = 37). A read family is a set of duplicate reads with the same start and end co-ordinates, which serve as an endogenous barcode[5]. Boxplots represent median, bottom and upper quartiles, and the whiskers correspond to 1.5× IQR. **(c)** Boxplots show the number of reads present at each stage of the Pointy pipeline for PGDX plasma WGS samples (n = 37). Details on each of the filter steps are outlined in the Methods. Boxplots

represent median, bottom and upper quartiles, and the whiskers correspond to 1.5× IQR. Source data are provided as a Source Data file. SNP, single nucleotide polymorphism.

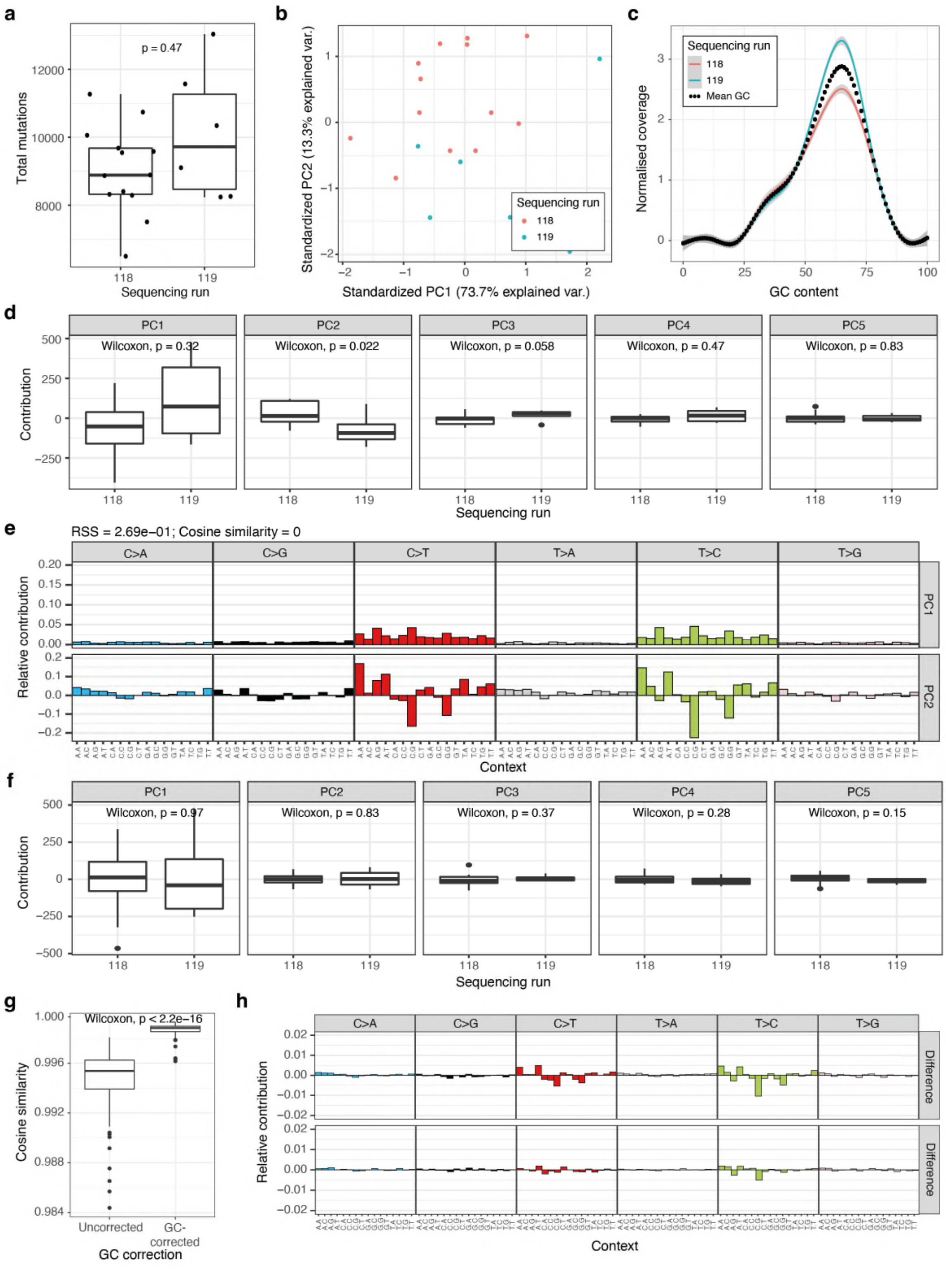


**Supplementary Fig. 3. GC normalization in control samples.** (**a**) Healthy control samples (n = 19) from the PGDX cohort were sequenced in two batches on the same

sequencer (run 118, n = 13; run 119, n = 6). Inter-batch differences in the total number of mutant reads per sample were compared using a two-sided Wilcoxon test. Boxplots represent median, bottom and upper quartiles, and whiskers correspond to 1.5× interquartile range (IQR). (**b**) PCA of raw SBS profiles of healthy samples (n = 19) showed clustering by sequencing run. (**c**) The GC content per bin is shown for the same healthy samples (n = 19), colored by batch. The average GC content profile across both batches is shown with a black dotted line, against which all samples are normalized (Methods). The gray shaded area indicates the 95% confidence interval of the fitted model. (**d**) Boxplots of each PC between sequencing runs 118 and 119 without GC-correction. Two-sided Wilcoxon tests were used. Boxplots represent median, bottom and upper quartiles, and whiskers correspond to 1.5× interquartile range (IQR). PC1, principal component 1; PC2, principal component 2. (**e**) SBS profiles of PC1 and PC2 are shown, indicating that PC2 is primarily composed of SBS contexts at the extremes of GC-content. RSS, residual sum of squares. (**f**) Following GC-bias correction, there was no significant difference in any PC. Two-sided Wilcoxon tests were used. Boxplots represent median, bottom and upper quartiles, and whiskers correspond to 1.5× interquartile range (IQR). (**g**) The cosine similarity between the mean SBS profile of healthy samples from each batch (118 vs. 119) was compared with and without GC-correction, using bootstrapping with 100 iterations. GC-corrected samples showed significantly greater cosine similarity ($P < 2.2 \times 10^{-16}$, two-sided Wilcoxon test). Boxplots represent bootstrapped median, bottom and upper quartiles, and whiskers correspond to 1.5× IQR. Points indicate outliers. (**h**) The differences in SBS profiles between each batch (118 vs. 119, n = 19) are shown for uncorrected (upper) and GC-corrected (lower) data. Source data are provided as a Source Data file.

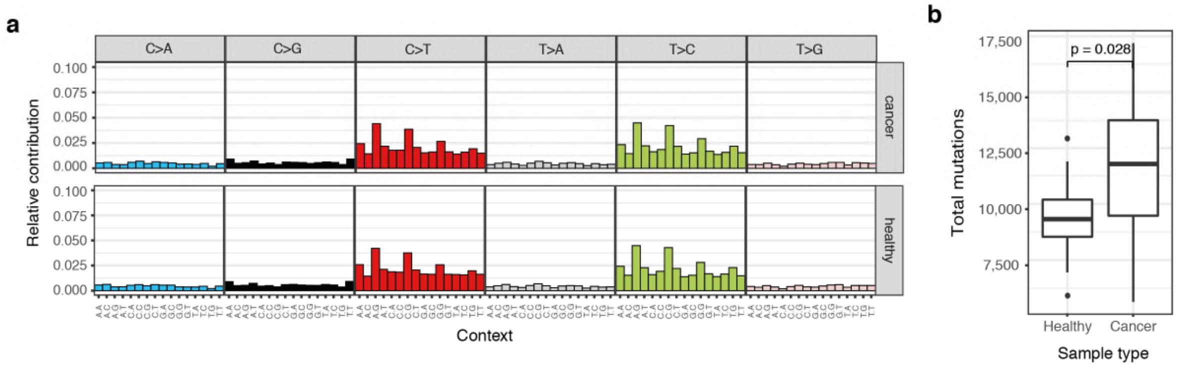


**Supplementary Fig. 4. Comparison of WGS mutation profiles following GC-correction. (a)** Aggregated 96-SBS profiles for healthy (n = 19) and CRC (n = 16) plasma WGS samples showed a cosine similarity of >0.99. **(b)** Boxplots comparing the total number of mutant reads (prior to background-subtraction) for healthy individuals (n = 19) vs. patients with stage IV CRC (n = 16). A two-tailed Wilcoxon test was used. Boxplots represent median, bottom and upper quartiles, and whiskers correspond to 1.5× interquartile range (IQR). Points indicate outliers. Source data are provided as a Source Data file.

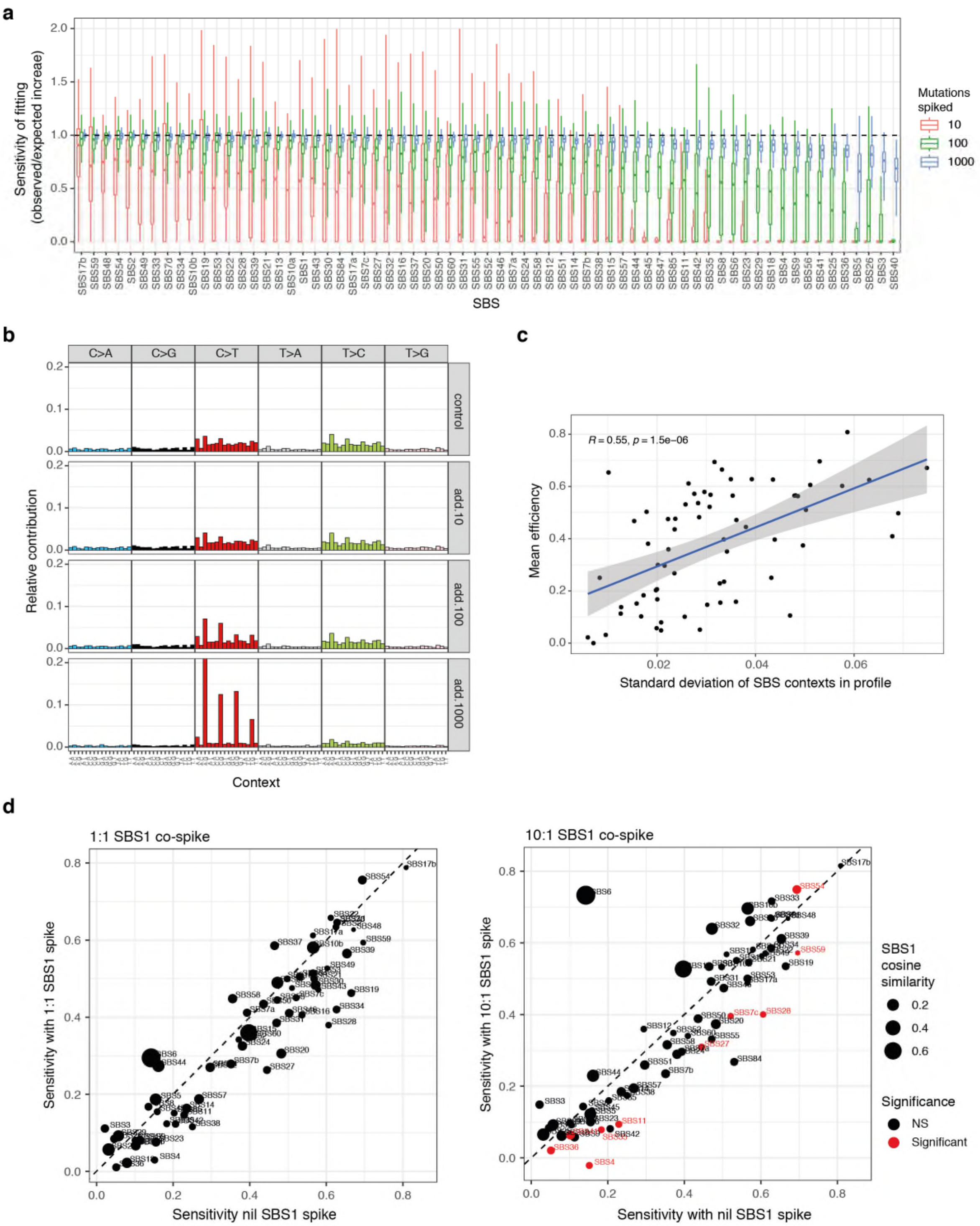


**Supplementary Fig. 5. *In silico* signature spike experiment to assess signature fitting sensitivity.** (**a**) Using the PGDX control plasma WGS samples (n = 19), an experiment of *in silico* signature spike-in and assessment of signature fitting sensitivity

was performed. Signature fitting sensitivity was defined as the ratio of the observed vs. expected increase in signature following spiking in known signatures to a randomly selected control SBS profile, with background-subtraction (Supplementary Methods). Either 10, 100 or 1,000 mutations belonging to each signature were spiked in during each iteration. 100 iterations were used. Boxplots represent median, bottom and upper quartiles, and whiskers correspond to 1.5× interquartile range (IQR). Red boxplots (left) indicate 10 spiked mutations; green boxplots (middle) indicate 100 spiked mutations; blue boxplots (right) indicate 1,000 spiked mutations. (**b**) Example SBS profiles for a mean healthy control profile (generated from 19 healthy controls), followed by spike-in of 10, 100 or 1,000 mutations belonging to SBS1 (Supplementary Methods). (**c**) The standard deviation of the proportions of each SBS context within each signature was used as a measure of flatness vs. spikiness of each signature. Signature spikiness showed significant positive correlation with mean fitting sensitivity ($p = 1.6 \times 10^{-11}$, two-sided Pearson correlation). The gray shaded area indicates the 95% confidence interval of the fitted linear model. (**d**) Co-spike experiment of SBS1 plus each signature at a ratio of 1:1 (left panel) or 10:1 (right panel) into a mean control SBS profile (generated from 19 healthy control plasma samples), with 100 iterations. Baseline spike in sensitivity using 10 mutations is shown on the x-axis, and sensitivity with either 1:1 (left panel) or 10:1 (right panel) SBS1-co-spike is shown on the y-axis. Signatures with zero mutations fitted with nil SBS1 spike-in are not shown. Data point size is proportional to the cosine similarity of that signature to SBS1. Signatures with a significant difference in sensitivity (following Benjamini-Hochberg correction) compared to the nil spike-in setting are highlighted in red. The dashed diagonal line indicates x = y. NS, non-significant. Source data are provided as a Source Data file.

**a**

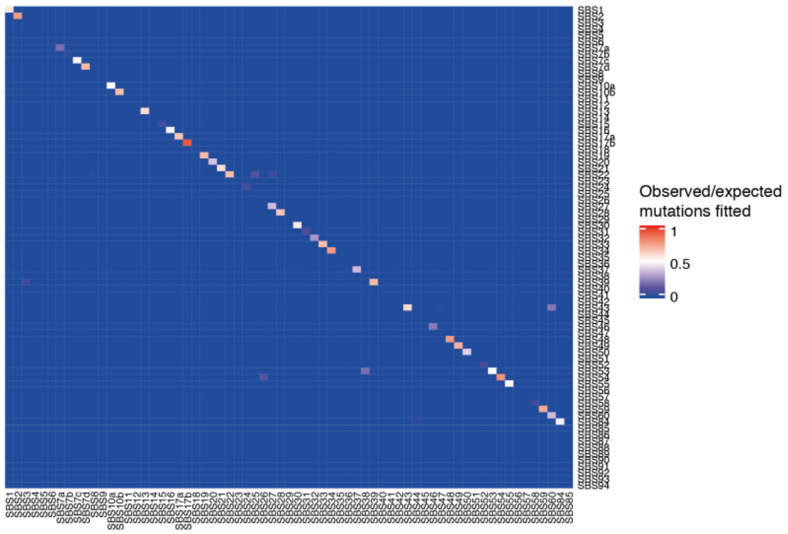
Observed/expected
mutations fitted
1
0.5
0

**b**

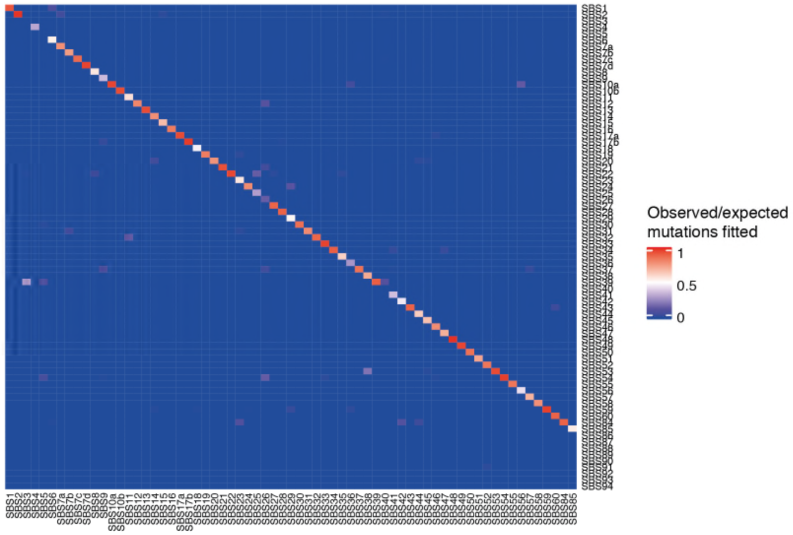
Observed/expected
mutations fitted
1
0.5
0

**c**

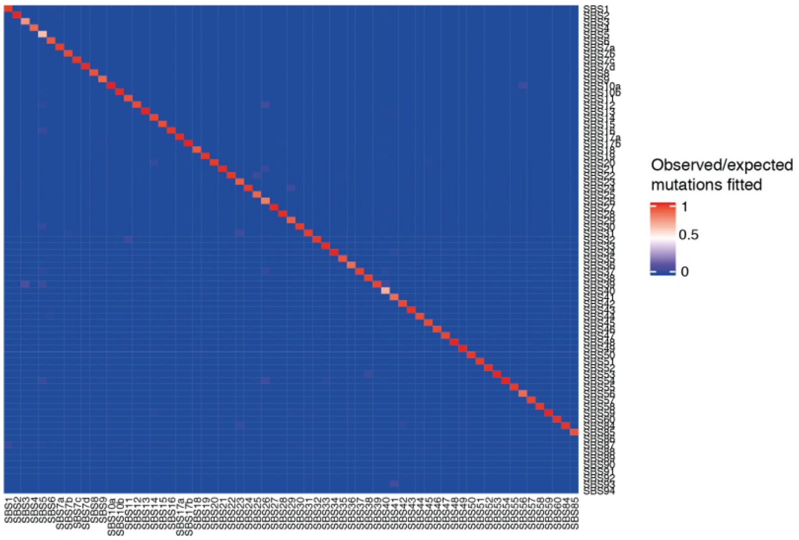
Observed/expected
mutations fitted
1
0.5
0

**Supplementary Fig. 6. Off-target signature fitting analysis.** *In silico* signature spike-in and assessment of signature fitting specificity. Using a mean-averaged single base substitution (SBS) mutation profile generated from 19 healthy control plasma samples from the PGDX cohort as a background, fixed doses of reference signatures were spiked in with (**a**) 10, (**b**) 100 and (**c**) 1,000 mutations. Signature spiking was performed with 100 iterations. Each panel shows a matrix of signature fitting sensitivity for pairwise spike-in. Signature fitting sensitivity is defined as the ratio of the observed vs. spiked in mutations. Blue indicates zero off-target signature fitting, whereas red indicates entirely off-target fitting. The diagonal line contains on-target signature fitting (i.e., SBS1 observed/expected, with SBS1 spiked-in). Source data are provided as a Source Data file.

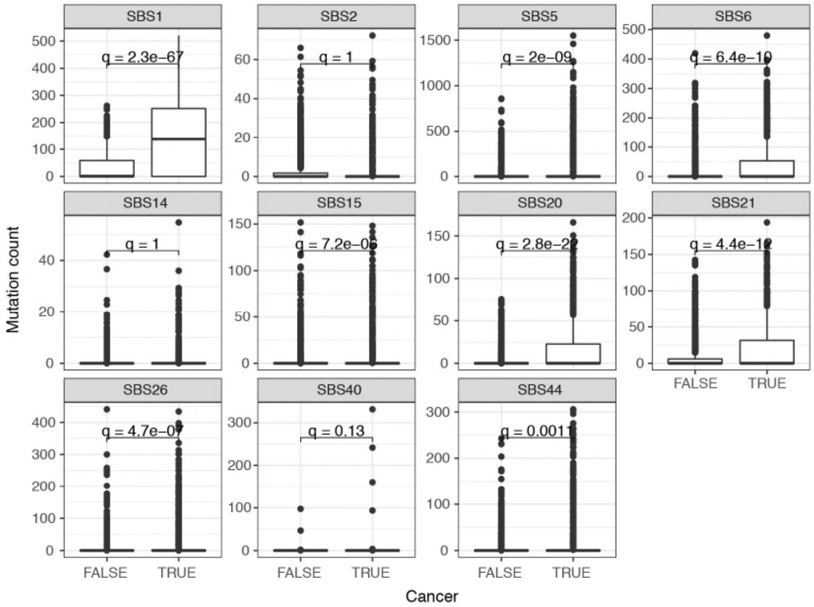


**Supplementary Fig. 7. Signature profile comparison using 10M reads, performed iteratively.** Using 16 PGDX plasma samples from individuals with CRC and 19 samples from healthy individuals, we tested whether differences in signature profile observed were stochastic due to downsampling sequencing reads. Therefore, we iteratively downsampled data to 10M reads 50 times, which showed that differences in signature contribution between cancer and healthy samples remain significant with repeated downsampling. One-tailed Wilcoxon tests were performed, and adjusted p-values (q) are shown. Boxplots represent the median, upper and lower quartiles and whiskers indicate 1.5 x IQR. Points indicate outliers. Source data are provided as a Source Data file.

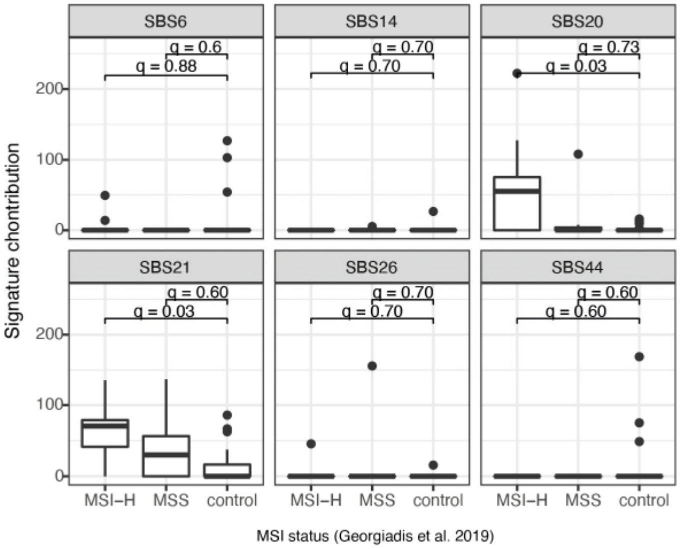


**Supplementary Fig. 8. Comparison of MSI signature contributions.** The contribution of MSI signatures in plasma was compared between patients classified as MSI-H (n = 9), MSS (n = 17) and healthy individuals (n = 19) from Georgiadis et al.[2]. Patients with MSS CRC had similar contributions of MSI signatures as healthy individuals ($p > 0.05$, one-sided Wilcoxon test), whereas patients with MSI-H CRC had significantly greater contributions of SBS20 and SBS21 compared to healthy ($p = 0.03$, one-sided Wilcoxon test). Boxplots represent the median, upper and lower quartiles and whiskers indicate 1.5 x IQR. Points represent outliers. Source data are provided as a Source Data file. MSI, microsatellite instability; MSI-H, microsatellite instability-high; MSS, microsatellite stable.

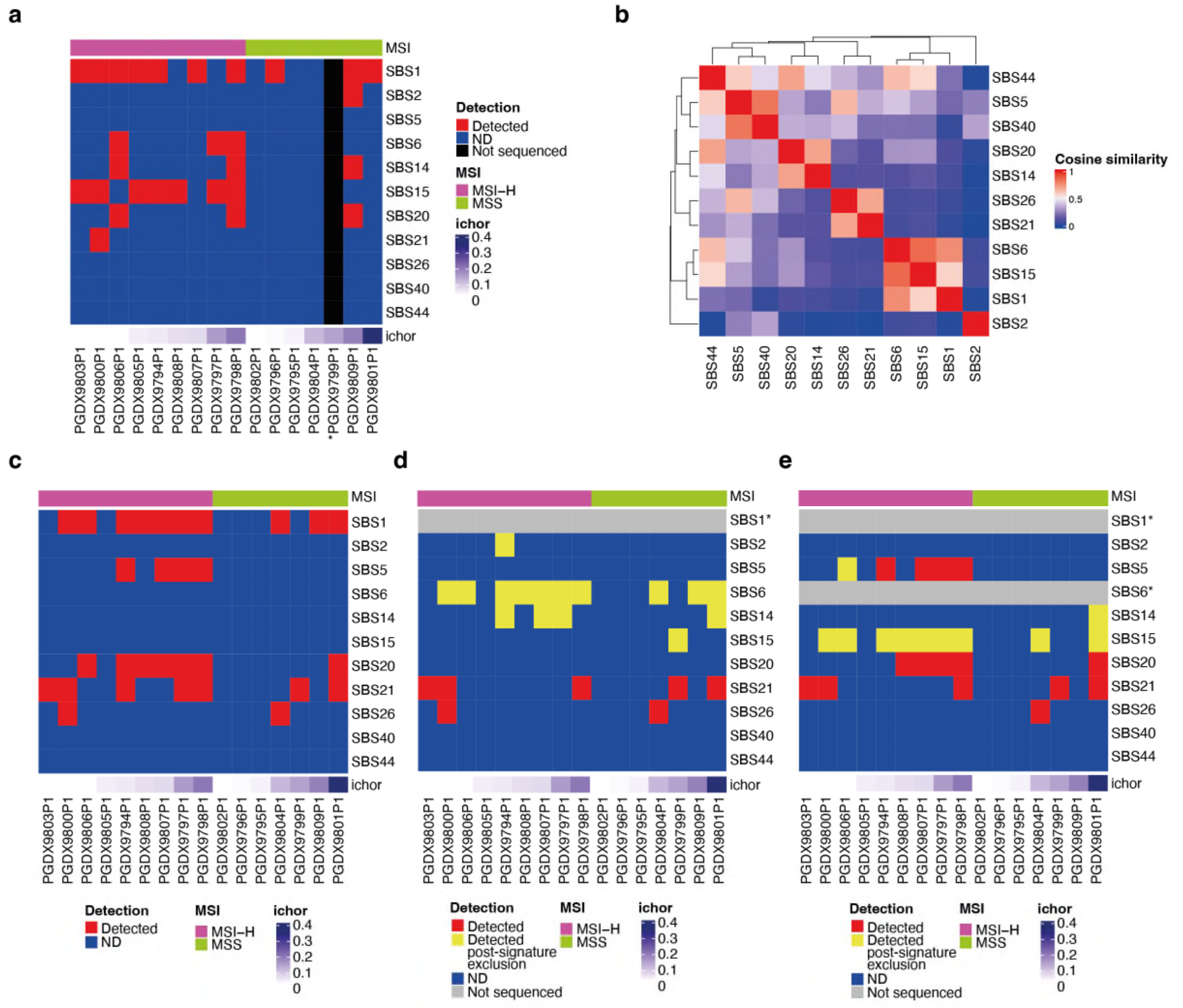


**Supplementary Fig. 9. Comparison of signatures identified by Pointy vs. targeted sequencing.** (**a**) Signatures identified using targeted sequencing of plasma samples in the CRC cohort (n = 15, Methods). The sample (n = 1) that was not sequenced with targeted sequencing is indicated in black. Fitted signatures are indicated in red; non-detected signatures are indicated in blue. (**b**) Cosine similarity matrix between 11 signatures relevant for CRC, clustered by similarity. Publicly available signatures[1] were used for clustering. Red indicates high cosine similarity, blue indicates low cosine similarity. (**c**) Heatmap of selected signatures detected in CRC plasma samples with 95% specificity (n = 16, Methods). Detected signatures are indicated in red, non-detected samples are shown in blue. Samples are annotated with ichorCNA ctDNA fraction and microsatellite instability status[2]. (**d**) Signature detection heatmap, similar to (c), except

with SBS1 excluded from signature fitting. Signatures that became detected are indicated in yellow. Gray indicates the excluded signature. (**e**) Signature detection heatmap, similar to (c), except with both SBS1 and SBS6 excluded from signature fitting. Gray indicates the excluded signatures. Source data are provided as a Source Data file. MSI, microsatellite instability; MSI-H, microsatellite instability-high; MSS, microsatellite stable.

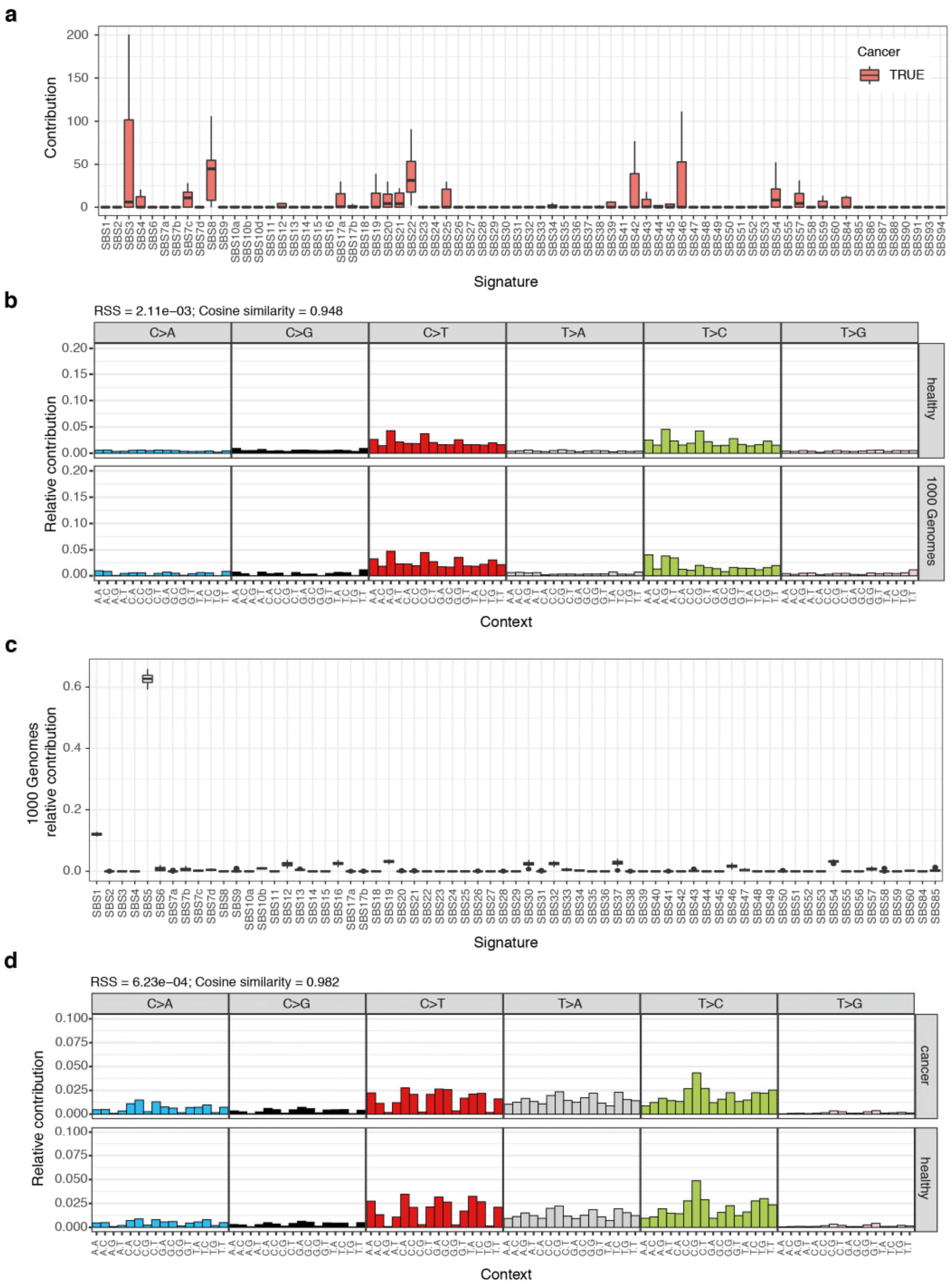


**Supplementary Fig. 10. SBS profiles following SNP-subtraction data. (a)** Signature fitting was performed on patient samples from the PGDX cohort (n = 16 individuals with CRC) following SNP-subtraction. The number of mutations attributed to each signature

(after background subtraction relative to controls) are shown. Following SNP subtraction, SBS1´ (SNP-subtracted) and SBS5´ were no longer assigned mutations, compared to SNP-retained data (Fig. 2a). Instead, other signatures such as SBS3´, SBS8´ and SBS46´, were attributed mutations. Boxplots represent the median, upper and lower quartiles and whiskers indicate 1.5 x IQR. **(b)** The aggregated SBS profile is shown for healthy samples (n = 16) from the PGDX cohort (with SNPs included), compared with the aggregated mutations from the 1000 Genomes database (k1g). The 1000 Genomes database was downloaded, annotated with SBS contexts, and all mutations were combined to generate an aggregated SBS profile. **(c)** The k1g mutation profile was bootstrapped 50 times and signatures were iteratively fitted. Signature fitting to the k1g profile showed that the majority of mutations are attributable to aging signatures (SBS1 and SBS5), and thus subtraction of k1g profile from plasma mutation data may bias signature profiling. Boxplots represent bootstrapped median, bottom and upper quartiles of the bootstrapped data, and the whiskers correspond to 1.5× IQR. **(d)** SBS profiles are shown for both aggregated healthy (n = 19) and CRC samples (n = 16) from the PGDX cohort following SNP-subtraction using the k1g database. Following SNP-subtraction, cancer samples vs. healthy samples showed a cosine similarity of 0.982, compared to 0.999 with SNPs included (Supplementary Fig. 4a). Source data are provided as a Source Data file.

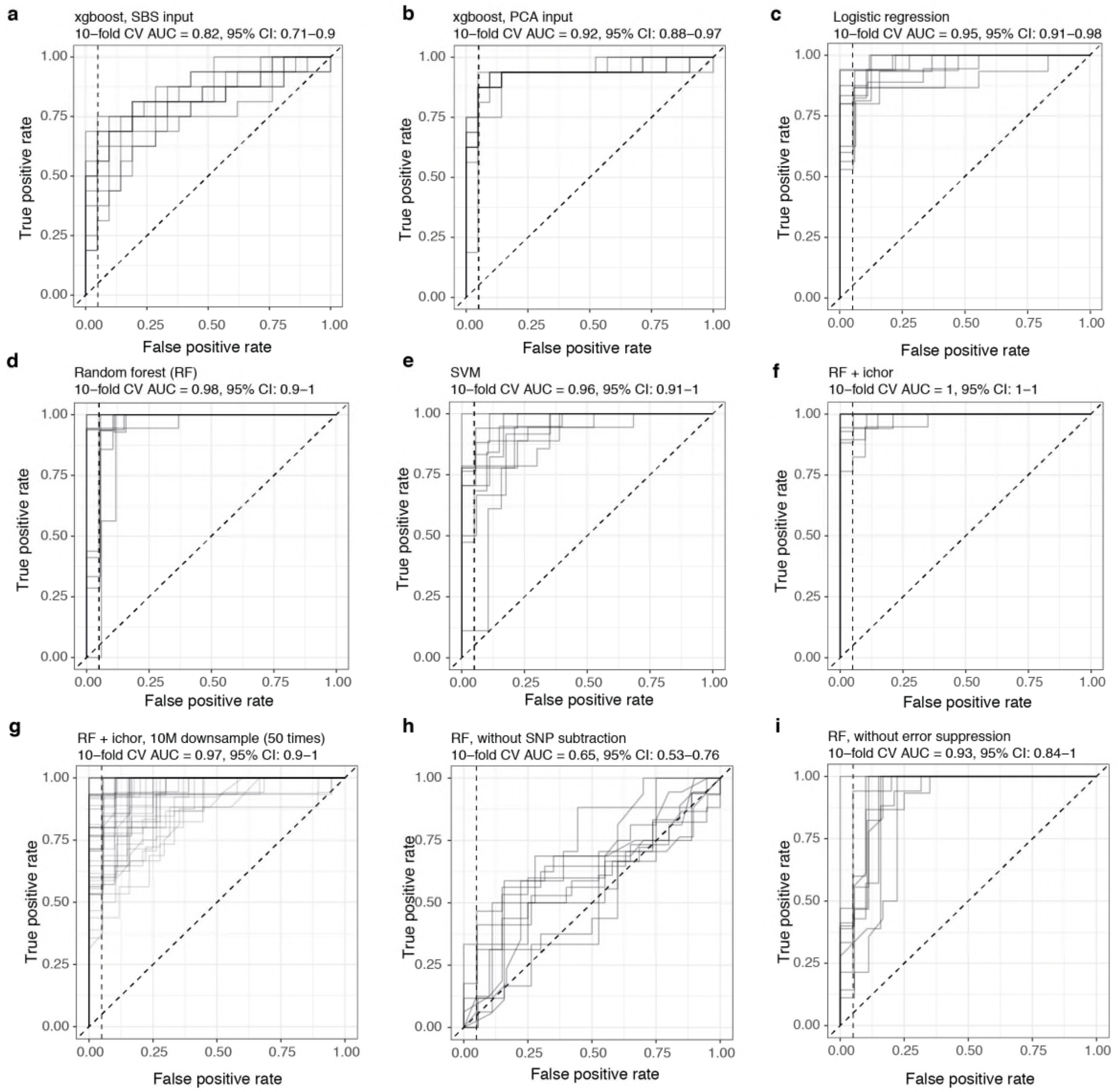


**Supplementary Fig. 11. Performance comparison of data processing steps and machine learning models.** 0.3x plasma WGS data from healthy individuals (n = 19) and patients with stage IV CRC (n = 16) were used to test different machine learning models for classification using point mutations and copy number from ichorCNA as input (Methods). (**a**) Classification performance using xgboost with raw SBS mutation matrix input following SNP-subtraction. Nested 10-fold cross-validation was performed, repeated 10 times. AUC, area under the curve. (**b**) Classification performance using xgboost with PCA-transformed input following SNP-subtraction. **(c)** Classification

performance using a logistic regression model with PCA-transformed input following SNP-subtraction. **(d)** Classification performance using a random forest model with PCA-transformed input following SNP-subtraction. **(e)** Classification performance using a support-vector machine model with PCA-transformed input following SNP-subtraction. (**f**) Classification performance using a random forest model with PCA-transformed input following SNP-subtraction, with ichorCNA ctDNA fractions included in model training. (**g**) To test the effect of downsampling data to 10M reads, using a random forest model, sequencing data were iteratively downsampled (50 iterations) to 10M reads and classified into cancer (n = 16) vs. healthy (n = 19). (**h**) Classification performance using a random forest model with PCA-transformed, without SNP-subtraction. (**i**) Classification performance using a random forest model with PCA-transformed data, with SNP-subtraction, but without error-suppression (see Supplementary Fig. 11d for error-suppressed data). Source data are provided as a Source Data file. AUC, area under the curve; CV, cross-validation; PCA, principal component analysis; RF, random forest.

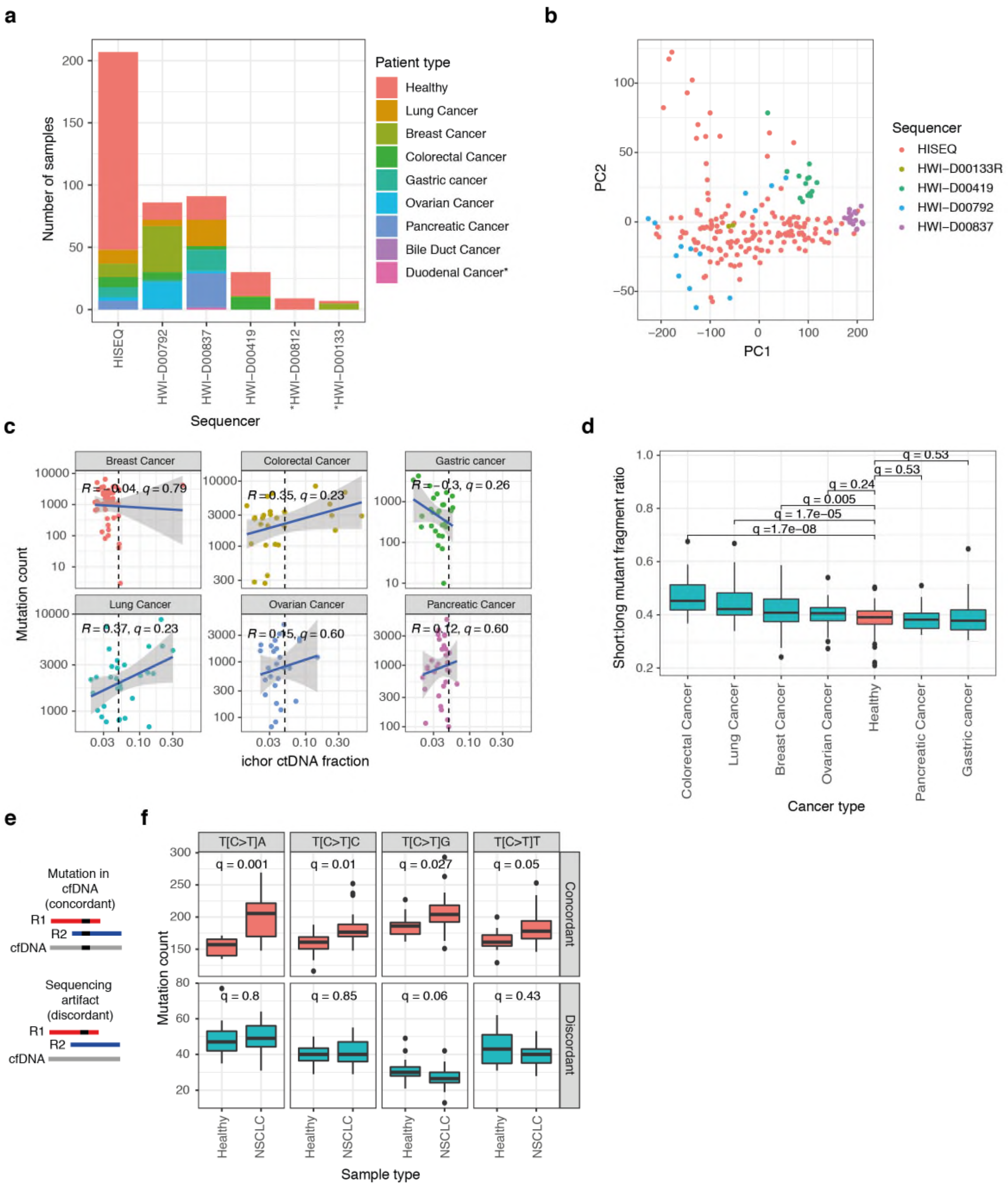


**Supplementary Fig. 12. Signature and fragmentation profiling of the DELFI cohort.** (**a**) Samples were sequenced across multiple sequencers in the DELFI study[3] (n = 423). The number of samples per sequencer is shown by cancer type. For classification analyses, samples were controlled against healthy individuals sequenced on the same sequencer, based on sequencer ID. *Sequencers HWI-D00812, HWI-D00133R were

excluded due to limited sample number and/or containing healthy samples only (n = 16); duodenal cancer samples and bile duct cancer samples were excluded due to limited numbers (n = 2). (**b**) Principal component analysis (PCA) of 96-SBS mutation profile of healthy individuals from the DELFI cohort (n = 208). Data points are colored by sequencer. PC, principal component. **(c)** Two-sided Pearson correlations between total number of mutations and ctDNA fraction (determined by ichorCNA) for all patients in the DELFI cohort (n = 199). BH-corrected p-values (q) are shown. The vertical dashed line indicates the 95% specificity for detection using ichorCNA. The gray shaded area indicates the 95% confidence interval of the fitted linear model. **(d)** The ratio of short:long (threshold = 150bp) mutant fragments was compared between patients (n = 199) and healthy controls (n = 206). Two-tailed Wilcoxon tests were used, and p-values (q) were BH corrected. Boxplots represent the median, upper and lower quartiles and whiskers indicate 1.5 x IQR. Points represent outliers. **(e)** Mutations in the overlapping region of a paired-end sequencing read can be either discordant or concordant. True mutations (or PCR artifact) would be present in both R1 and R2. Discordant mutations are unlikely to be biological signal, and may be used to assess sequencing noise. **(f)** For the most prevalent SBS contexts of SBS2 (which comprise 97.8% of the signature), concordant and discordant mutations were compared between healthy individuals (n = 21) and patients with NSCLC (n = 19) belonging to one sequencer ID (HWI-D00837). Two-tailed Wilcoxon tests were used, and p-values (q) were BH corrected. Boxplots represent the median, upper and lower quartiles and whiskers indicate 1.5 x IQR. Points represent outliers. Source data are provided as a Source Data file.

**a**

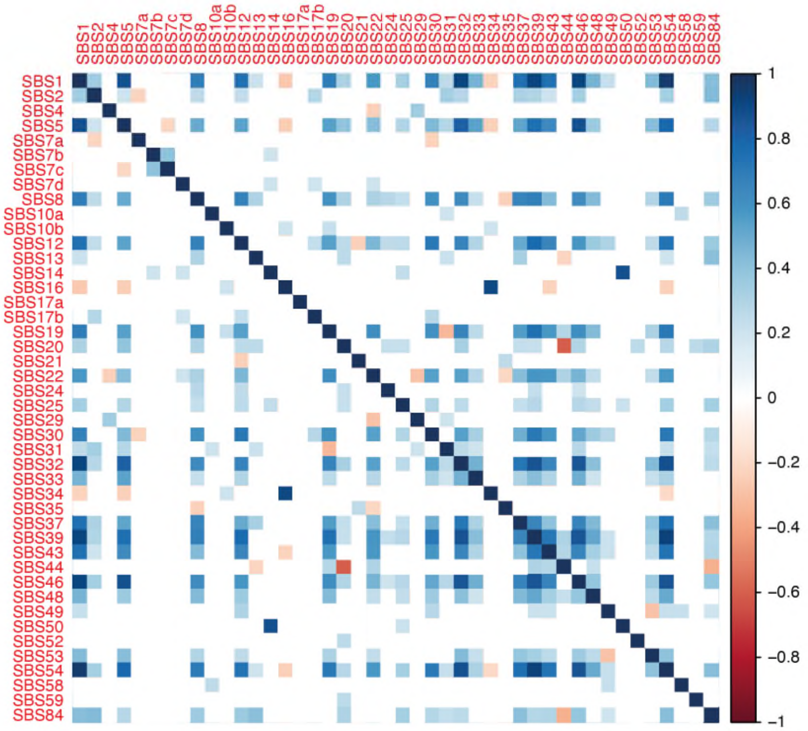
SBS1
SBS2
SBS4
SBS5
SBS7a
SBS7b
SBS7c
SBS7d
SBS8
SBS10a
SBS10b
SBS12
SBS13
SBS14
SBS16
SBS17a
SBS17b
SBS19
SBS20
SBS21
SBS22
SBS24
SBS25
SBS29
SBS30
SBS31
SBS32
SBS33
SBS34
SBS35
SBS37
SBS39
SBS43
SBS44
SBS46
SBS48
SBS49
SBS50
SBS52
SBS53
SBS54
SBS58
SBS59
SBS84
1
0.8
0.6
0.4
0.2
0
−0.2
−0.4
−0.6
−0.8
−1

**b**

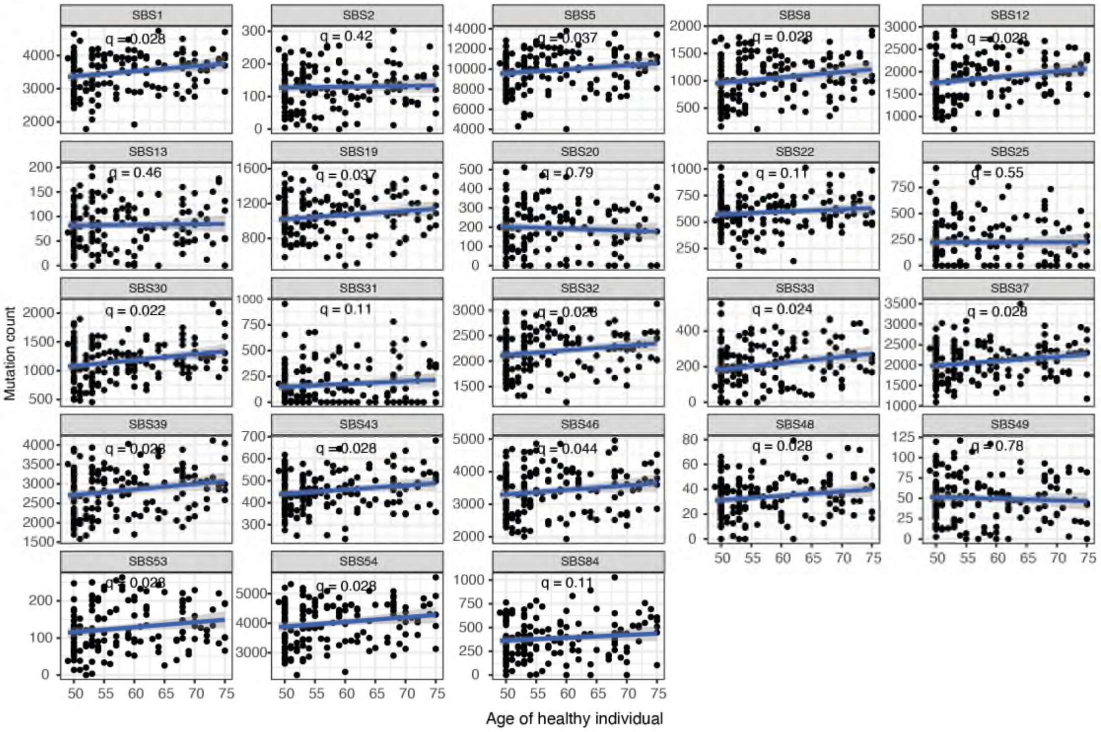
SBS1
q = 0.028
SBS2
q = 0.42
SBS5
q = 0.037
SBS8
q = 0.028
SBS12
q = 0.028
SBS13
q = 0.46
SBS19
q = 0.037
SBS20
q = 0.79
SBS22
q = 0.11
SBS25
q = 0.55
SBS30
q = 0.022
SBS31
q = 0.11
SBS32
q = 0.028
SBS33
q = 0.024
SBS37
q = 0.028
SBS39
q = 0.028
SBS43
q = 0.028
SBS46
q = 0.044
SBS48
q = 0.028
SBS49
q = 0.78
SBS53
q = 0.028
SBS54
q = 0.028
SBS84
q = 0.11
Mutation count
Age of healthy individual
50 55 60 65 70 75

**Supplementary Fig. 13. Correlation between age and signature contributions in SNP retained data from healthy individuals. (a)** SBS signature contributions using SNP-retained data from 159 healthy individuals sequenced on the same machine (HISEQ) from the DELFI cohort were correlated. Data were processed as before, except with 50M reads to maximize sensitivity for physiological signatures. Only signature correlations with a significance of BH-corrected p-value $< 0.05$ are shown in color (two-sided Pearson correlation). Red indicates positive correlation, blue indicates negative correlation. (**b**) Signatures that were identified to be SBS1-correlated were correlated against chronological age in the same cohort of healthy individuals ($n = 159$). SNP-retained data were used. One-sided Pearson correlations were calculated for each SBS and BH correction for multiple testing was performed. The gray shaded area indicates the 95% confidence interval of the fitted linear model. Source data are provided as a Source Data file.

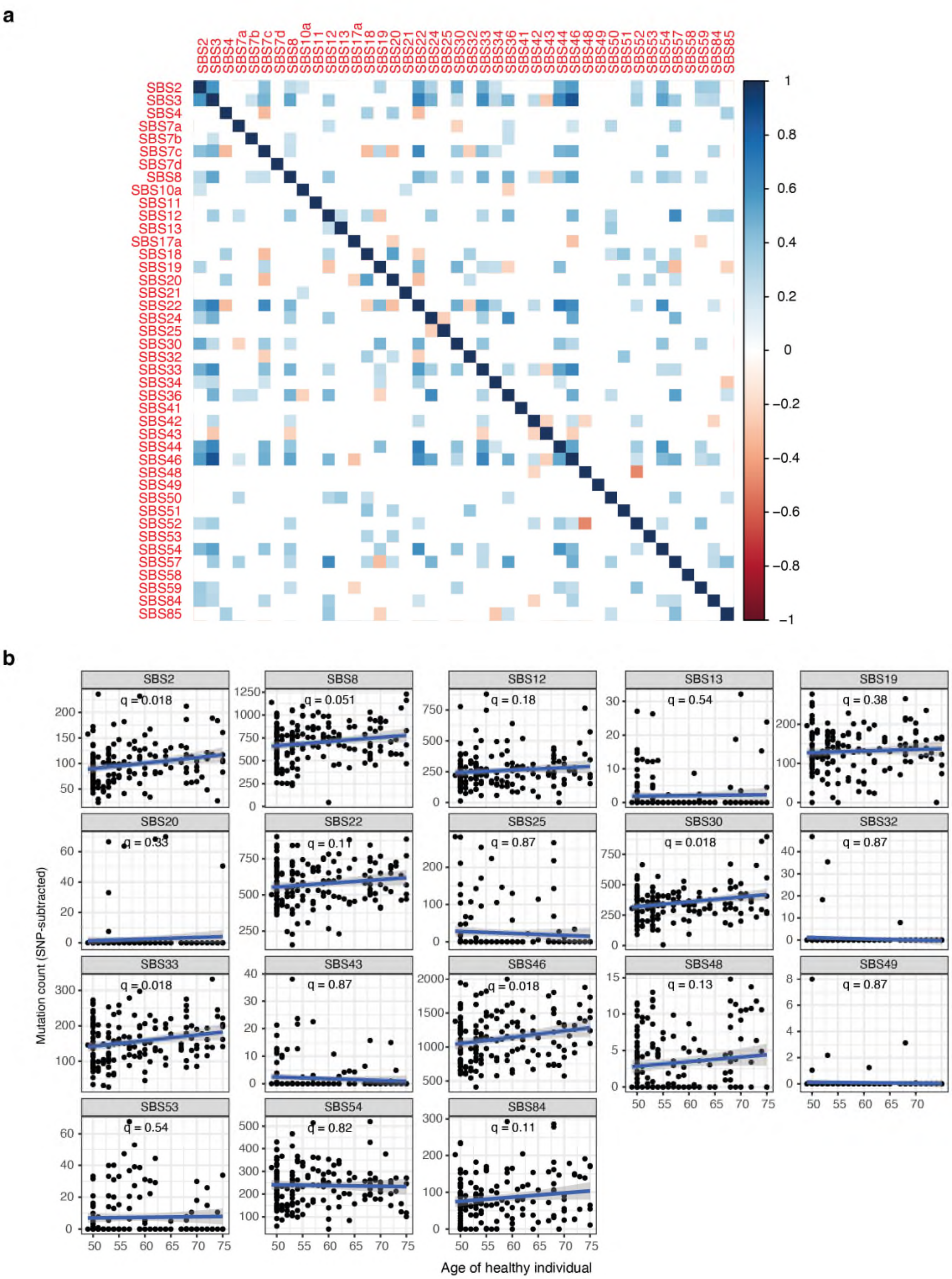
a
SBS2 SBS3 SBS4 SBS7a SBS7b SBS7c SBS7d SBS8 SBS10a SBS11 SBS12 SBS13 SBS17a SBS18 SBS19 SBS20 SBS21 SBS22 SBS24 SBS25 SBS30 SBS32 SBS33 SBS34 SBS36 SBS41 SBS42 SBS43 SBS44 SBS46 SBS48 SBS49 SBS50 SBS51 SBS52 SBS53 SBS54 SBS57 SBS58 SBS59 SBS84 SBS85
1
0.8
0.6
0.4
0.2
0
−0.2
−0.4
−0.6
−0.8
−1
b
SBS2 q = 0.018
SBS8 q = 0.051
SBS12 q = 0.18
SBS13 q = 0.54
SBS19 q = 0.38
SBS20 q = 0.33
SBS22 q = 0.11
SBS25 q = 0.87
SBS30 q = 0.018
SBS32 q = 0.87
SBS33 q = 0.018
SBS43 q = 0.87
SBS46 q = 0.018
SBS48 q = 0.13
SBS49 q = 0.87
SBS53 q = 0.54
SBS54 q = 0.82
SBS84 q = 0.11
Mutation count (SNP-subtracted)
Age of healthy individual

**Supplementary Fig. 14. Correlation between age and signature contributions in SNP-subtracted data from healthy individuals. (a)** SBS signature contributions using SNP-subtracted data from 159 healthy individuals sequenced on the same machine (HISEQ) from the DELFI cohort were correlated. Data were processed as before, except with 50M reads to maximize sensitivity for physiological signatures. Only signature correlations with a significance of BH-corrected p-value < 0.05 are shown in color (two-sided Pearson correlation). Red indicates positive correlation, blue indicates negative correlation. (**b**) SNP-subtracted signatures that were identified to be SBS1-correlated in Supplementary Fig. 13a were correlated against chronological age in the same cohort of healthy individuals (n = 159). SNP-subtracted data were used, resulting in no mutations fitting to SBS1´ or SBS5´ (Supplementary Fig. 10). One-sided Pearson correlations were calculated for each SBS and BH correction for multiple testing was performed. The gray shaded area indicates the 95% confidence interval of the fitted linear model. Source data are provided as a Source Data file.

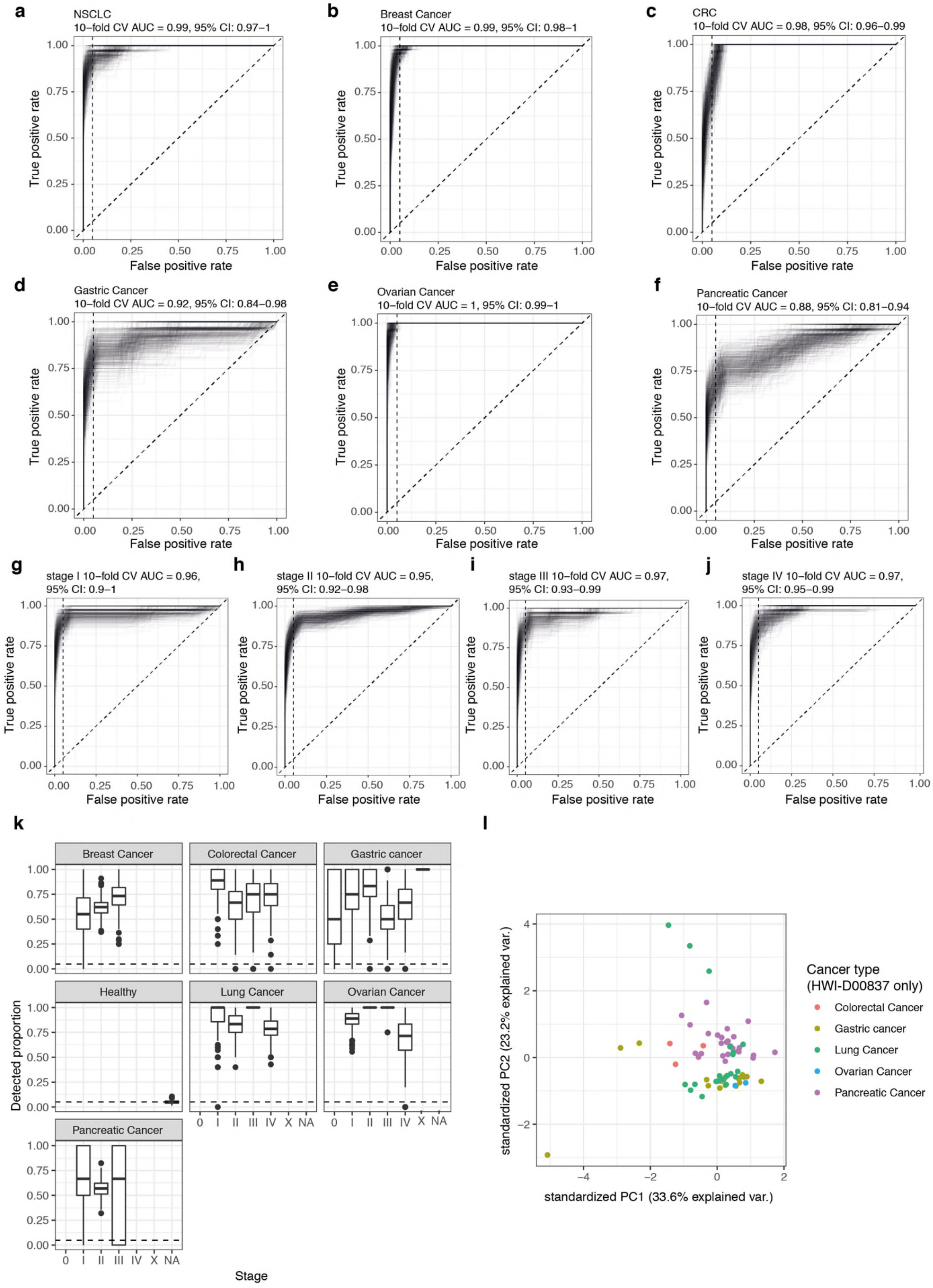

a
NSCLC
10–fold CV AUC = 0.99, 95% CI: 0.97–1
b
Breast Cancer
10–fold CV AUC = 0.99, 95% CI: 0.98–1
c
CRC
10–fold CV AUC = 0.98, 95% CI: 0.96–0.99
d
Gastric Cancer
10–fold CV AUC = 0.92, 95% CI: 0.84–0.98
e
Ovarian Cancer
10–fold CV AUC = 1, 95% CI: 0.99–1
f
Pancreatic Cancer
10–fold CV AUC = 0.88, 95% CI: 0.81–0.94
g
stage I 10–fold CV AUC = 0.96, 95% CI: 0.9–1
h
stage II 10–fold CV AUC = 0.95, 95% CI: 0.92–0.98
i
stage III 10–fold CV AUC = 0.97, 95% CI: 0.93–0.99
j
stage IV 10–fold CV AUC = 0.97, 95% CI: 0.95–0.99
True positive rate
False positive rate
k
Breast Cancer
Colorectal Cancer
Gastric cancer
Healthy
Lung Cancer
Ovarian Cancer
Pancreatic Cancer
Detected proportion
Stage
0 I II III IV X NA
l
standardized PC1 (33.6% explained var.)
standardized PC2 (23.2% explained var.)
Cancer type (HWI-D00837 only)
Colorectal Cancer
Gastric cancer
Lung Cancer
Ovarian Cancer
Pancreatic Cancer

**Supplementary Fig. 15. Classification performance using Pointy on the DELFI data set.** Cancer detection performance using a random forest (RF) model in the DELFI data set was assessed across all cancer stages (n = 199), using 10-fold nested cross-validation using a random forest model (500 iterations). (**a**) stage I-IV non-small cell lung cancer (NSCLC, n = 37). (**b**) stage I-III breast cancer (n = 48). (**c**) stage I-IV CRC (n = 27). (**d**) stage I-IV and X gastric cancer (n = 27). (**e**) stages I, III and IV ovarian cancer (n = 26). (**f**) stage I-III pancreatic cancer (n = 34). (**g-j**) Detection rates were next assessed by stage: stage I (n = 41), stage II (n = 86), stage III (n = 33), stage IV (n = 36). (**k**) Detection rates by stage and cancer type for all patients and all stages (n = 199), using a 95% specificity threshold for detection. Healthy samples (n = 206) are included as stage = NA. Boxplots represent bootstrapped median, bottom and upper quartiles, and whiskers correspond to 1.5× IQR. Points indicate outliers. **(l)** PCA of plasma SBS mutation profiles from patients all sequenced on the same sequencer (HWI-D00837, n = 70). This shows separation of samples by cancer type in PC1 and PC2, which may enable classification by cancer type. Source data are provided as a Source Data file. AUC, area under the curve; CV, cross-validation; PC, principal component.

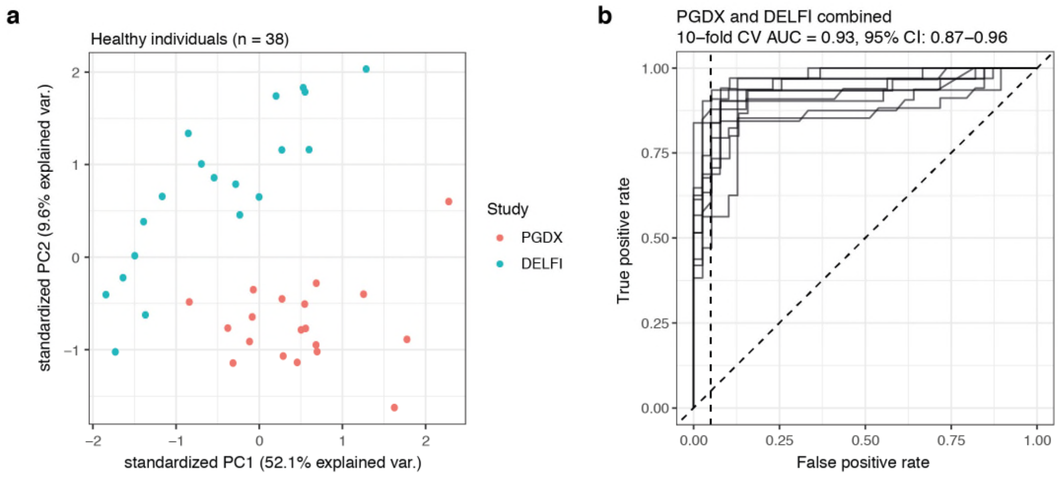


**Supplementary Fig. 16. Batch effects across studies and cancer detection across cohorts.** (**a**) PCA of 96-SBS profiles of healthy individuals (n = 38) from each of the datasets used shows evidence of batch effect (PGDX, red; DELFI, blue). All healthy individuals were included from the PGDX cohort (n = 19), and an equal number were randomly selected from the DELFI cohort (n = 19), sampled with a fixed seed. Data downsampled to 10M reads were used. (**b**) To assess the generalizability of Pointy across cohorts, samples were pooled between the two studies in an equal number, i.e., n = 19 healthy individuals and n = 16 CRC from each cohort. To generate an equal number of samples from the DELFI cohort, sampled with a fixed seed. Classification was performed using an RF model with 10-fold nested CV, using 10 iterations. Source data are provided as a Source Data file. AUC, area under the curve; CV, cross-validation.

## Supplementary References


1. Alexandrov, L. B. *et al.* The repertoire of mutational signatures in human cancer. *Nature* **578**, 94–101 (2020).
2. Georgiadis, A. *et al.* Noninvasive detection of microsatellite instability and high tumor mutation burden in cancer patients treated with PD-1 blockade. *Clin. Cancer Res.* **25**, 7024–7034 (2019).
3. Cristiano, S. *et al.* Genome-wide cell-free DNA fragmentation in patients with cancer. *Nature* **570**, 385–389 (2019).
4. Adalsteinsson, V. A. *et al.* Scalable whole-exome sequencing of cell-free DNA reveals high concordance with metastatic tumors. *Nat. Commun.* **8**, 1324 (2017).
5. Hoang, M. L. *et al.* Genome-wide quantification of rare somatic mutations in normal human tissues using massively parallel sequencing. *Proc. Natl. Acad. Sci.* **113**, 9846–9851 (2016).
6. Alexandrov, L. B., Jones, P. H., Wedge, D. C., Sale, J. E. & Peter, J. Clock-like mutational processes in human somatic cells. *Nat. Genet.* **47**, 1402–1407 (2015).
