# Supplementary material for: Genome-wide mutational signatures in low-coverage whole genome sequencing of cell-free DNA
Source: Nat Commun. 2022 Aug 23;13:4953. doi: 10.1038/s41467-022-32598-1 (PMC9399180; doi:10.1038/s41467-022-32598-1)
Supplement: Supplementary file 4 — Reporting Summary [file 41467_2022_32598_MOESM4_ESM.pdf]

nature research

Corresponding author(s): Dr Luis Diaz

Last updated by author(s): Jul 28, 2022

Reporting Summary

Nature Research wishes to improve the reproducibility of the work that we publish. This form provides structure for consistency and transparency in reporting. For further information on Nature Research policies, see our Editorial Policies and the Editorial Policy Checklist.

Statistics

For all statistical analyses, confirm that the following items are present in the figure legend, table legend, main text, or Methods section.

n/a	Confirmed	
☐	☒	The exact sample size (*n*) for each experimental group/condition, given as a discrete number and unit of measurement
☐	☒	A statement on whether measurements were taken from distinct samples or whether the same sample was measured repeatedly
☐	☒	The statistical test(s) used AND whether they are one- or two-sided<br>*Only common tests should be described solely by name; describe more complex techniques in the Methods section.*
☐	☒	A description of all covariates tested
☐	☒	A description of any assumptions or corrections, such as tests of normality and adjustment for multiple comparisons
☐	☒	A full description of the statistical parameters including central tendency (e.g. means) or other basic estimates (e.g. regression coefficient) AND variation (e.g. standard deviation) or associated estimates of uncertainty (e.g. confidence intervals)
☐	☒	For null hypothesis testing, the test statistic (e.g. *F*, *t*, *r*) with confidence intervals, effect sizes, degrees of freedom and *P* value noted<br>*Give P values as exact values whenever suitable.*
☒	☐	For Bayesian analysis, information on the choice of priors and Markov chain Monte Carlo settings
☒	☐	For hierarchical and complex designs, identification of the appropriate level for tests and full reporting of outcomes
☐	☒	Estimates of effect sizes (e.g. Cohen's *d*, Pearson's *r*), indicating how they were calculated

Our web collection on statistics for biologists contains articles on many of the points above.

Software and code

Policy information about availability of computer code

Data collection	Plasma samples were collected by PGDX or as part of the Cristiano et al. (2019) study.
Data analysis	Sequencing data were analyzed using the following tools: trimmomatic (v0.39), Bedtools (v2.28.0), BWA (v0.7.15), samtools (v1.7), Picard (v2.19.0), GATK (v3.8), annovar (v2018-04-16, using databases refSeq and dbSNP151), R (v4.1.2).<br><br>Base R packages used include: stats. Custom R scripts for data processing, signature profiling and cancer detection utilized the following packages: dplyr (v1.0.8), plyr (v1.8.7), MutationalPatterns (v2.0.0), xgboost (v1.5.2.1), randomForest (v4.6-14), e1071 (v1.7.9) cvAUC (v1.1.0), caret (v6.0-90). Data were visualized using ggplot2 (v3.3.5) and ggpubr (v0.4.0).<br><br>Code availability statement:<br>Code used in the Pointy pipeline is available for academic research purposes only at https://doi.org/10.5281/zenodo.6666951. Code is in a restricted-access repository; users are required to agree to the license terms and conditions prior to approval. We aim to respond to data access requests within 5 working days.

For manuscripts utilizing custom algorithms or software that are central to the research but not yet described in published literature, software must be made available to editors and reviewers. We strongly encourage code deposition in a community repository (e.g. GitHub). See the Nature Research guidelines for submitting code & software for further information.

Data

Policy information about availability of data

All manuscripts must include a data availability statement. This statement should provide the following information, where applicable:

- Accession codes, unique identifiers, or web links for publicly available datasets
- A list of figures that have associated raw data
- A description of any restrictions on data availability

Sequence data have been deposited at the European Genome-phenome Archive (EGA), which is hosted by the EBI and the CRG, under accession number EGAS00001006377 [https://ega-archive.org/studies/EGAS00001006377]. Sequence data from the Cristiano et al. [9] study were previously deposited at the EGA, under accession number EGAD00001005339 [https://ega-archive.org/datasets/EGAD00001005339]. Further information about EGA can be found on https://ega-archive.org "The European Genome-phenome Archive of human data consented for biomedical research" [46]. The sequencing data are available under restricted access to comply with patient consent for data sharing, access can be obtained by approval via their respective Data Access Committees via the EGA. Source data are provided as a Source Data file. The following public databases were used to annotate mutations: 1000 Genomes [23], RefSeq [39] and dbSNP 151 [40]. DELFI sequencing data are publicly available [9].

Field-specific reporting

Please select the one below that is the best fit for your research. If you are not sure, read the appropriate sections before making your selection.

☒ Life sciences ☐ Behavioural & social sciences ☐ Ecological, evolutionary & environmental sciences

For a reference copy of the document with all sections, see nature.com/documents/nr-reporting-summary-flat.pdf

Life sciences study design

All studies must disclose on these points even when the disclosure is negative.

Sample size	For this study, pre-existing datasets were used. We performed the following analyses to estimate the effect size that may be observed with the sample sizes available. In the PGDX cohort, with an alpha = 0.05, beta = 0.2, n = 37, we estimated that this analysis would be powered to detect a 25% difference in signature contribution between cases and controls, when considered in aggregate.<br><br>In the DELFI cohort, with an alpha = 0.05, beta = 0.2, n = 159, we estimated that for the analysis of healthy individuals, we would be powered to observe a Pearson correlation between aging signatures and chronological age of Pearson r >0.22.
Data exclusions	Samples with fewer than 90% of the sequencing reads of the target downsampled sequencing depth were excluded. These samples are listed in Supplementary Data 1.
Replication	The analyses of mutational signature profiling and cancer detection were performed first in the PGDX CRC cohort (Figs. 1-3), and in an independent, publicly-available dataset (Figs. 4 and 6). Nested 10-fold cross-validation (repeated 500 times) was performed for cancer detection in each cohort, and was repeated on a combined cohort of PGDX CRC patients, PGDX healthy individuals, DELFI CRC patients and DELFI healthy individuals.
Randomization	Randomization was not performed in this retrospective and non-interventional study.
Blinding	Blinded analysis was not performed in this study. However, to mitigate the risk of bias, cancer classification analyses were performed with 10-fold nested cross validation, repeated 500 times, carried out across independent cohorts.

Reporting for specific materials, systems and methods

We require information from authors about some types of materials, experimental systems and methods used in many studies. Here, indicate whether each material, system or method listed is relevant to your study. If you are not sure if a list item applies to your research, read the appropriate section before selecting a response.

Materials & experimental systems

n/a	Involved in the study	
☒	☐	Antibodies
☒	☐	Eukaryotic cell lines
☒	☐	Palaeontology and archaeology
☒	☐	Animals and other organisms
☐	☒	Human research participants
☒	☐	Clinical data
☒	☐	Dual use research of concern

Methods

n/a	Involved in the study	
☒	☐	ChIP-seq
☒	☐	Flow cytometry
☒	☐	MRI-based neuroimaging

Human research participants

Policy information about studies involving human research participants

Population characteristics	In this study, cfDNA WGS data were analyzed from a total of 215 patients and 227 healthy control individuals across two cohorts (Supplementary Data 1). For the initial cohort (PGDX), 16 patients with stage IV CRC provided plasma samples following written informed consent for research use as part of clinical trial NCT01876511. This protocol was approved by the Johns Hopkins Institutional Review Board [21,36]. Plasma samples from 21 healthy control individuals were procured through BioIVT [21].<br><br>We next studied 199 patients and 206 healthy control individuals from the DELFI9 dataset following approval from their Data Access Committee (DAC). Samples in this cohort were obtained under Institutional Review Board approved protocols, with informed consent from all participants for research use at participating institutions [9]. Patients with the following cancer types were included from the DELFI study: CRC (n = 27), gastric (n = 27), NSCLC (n = 37), ovarian (n = 26), breast (n = 48), pancreatic (n = 34). Only pre-treatment timepoints from the DELFI study were used. For this proof-of-concept study, no blinding or randomization were performed.
Recruitment	For the PGDX cohort: as described in Le et al., 2015 & Georgiadis et al., 2019. For the DELFI cohort: as described in Cristiano et al., 2019. Please see detail regarding recruitment and consent in text above.
Ethics oversight	For the PGDX cohort: as described in Le et al., 2015 & Georgiadis et al., 2019. For the DELFI cohort: as described in Cristiano et al., 2019. Please see detail regarding recruitment and consent in text above.

Note that full information on the approval of the study protocol must also be provided in the manuscript.
